# Characterization of Aluminum-Based-Surface Matrix Composites with Iron and Iron Oxide Fabricated by Friction Stir Processing

**DOI:** 10.3390/ma9070505

**Published:** 2016-06-23

**Authors:** Essam R. I. Mahmoud, Mahmoud M. Tash

**Affiliations:** 1 Mechanical Engineering, King Khalid University, Abha 61413, Saudi Arabia; emahoud@kku.edu.sa; 2 Welding and NDT Laboratory, Manufacturing Technology Department, Central Metallurgical Research and Development Institute (CMRDI), Cairo 11421, Egypt; 3 Industrial Engineering Program, Department of Mechanical Engineering, University of Prince Sattam bin Abdulaziz, AlKharj 11942, Saudi Arabia; 4 On Leave from the Mining, Petroleum and Metallurgical Engineering Department, Cairo University, Giza 12613, Egypt

**Keywords:** magnetization, aluminum, friction stir processing, surface composites, iron powder, Fe_3_O_4_

## Abstract

Surface composite layers were successfully fabricated on an A 1050-H24 aluminum plate by dispersed iron (Fe) and magnetite (Fe_3_O_4_) particles through friction stir processing (FSP). Fe and Fe_3_O_4_ powders were packed into a groove of 3 mm in width and 1.5 mm in depth, cut on the aluminum plate, and covered with an aluminum sheet that was 2-mm thick. A friction stir processing (FSP) tool of square probe shape, rotated at a rate of 1000–2000 rpm, was plunged into the plate through the cover sheet and the groove, and moved along the groove at a travelling speed of 1.66 mm/s. Double and triple passes were applied. As a result, it is found that the Fe particles were homogenously distributed in the whole nugget zone at a rotation speed of 1000 rpm after triple FSP passes. Limited interfacial reactions occurred between the Fe particles and the aluminum matrix. On the other hand, the lower rotation speed (1000 rpm) was not enough to form a sound nugget when the dispersed particles were changed to the larger Fe_3_O_4_. The Fe_3_O_4_ particles were dispersed homogenously in a sound nugget zone when the rotation speed was increased to 1500 rpm. No reaction products could be detected between the Fe_3_O_4_ particles and the aluminum matrix. The saturation magnetization (Ms) of the Fe-dispersed nugget zone was higher than that of the Fe_3_O_4_-dispersed nugget zone. Moreover, there were good agreement between the obtained saturation magnetization values relative to that of pure Fe and Fe_3_O_4_ materials and the volume content of the dispersed particles in the nugget zone.

## 1. Introduction

Aluminum alloys possess attractive properties, such as low density, high strength-to weight ratio, excellent corrosion resistance and a relatively low cost [1,2,3], which make them an optimum structural material for some special-use applications, such as magnetic refrigeration and kitchen utensils for induction heating. A major impediment that restricts their application is their poor magnetic properties [4]. This restriction can be removed if a magnetic layer is cladded on their surfaces or particles with appropriate magnetic properties are embedded in the aluminum matrix to form surface metal matrix composites (SMMCs) [5,6,7]. In these situations, a novel and interesting physical property, “magnetization”, is introduced into the aluminum alloys, in addition to their original excellent physical and mechanical properties. For this purpose, the selection of both suitable filler and matrix materials is very important. From a health point of view, pure aluminum will be the matrix material [8,9]. On the other hand, iron (Fe) and magnetite (Fe_3_O_4_) powders are strong candidates to be used for filling due to their good magnetic properties [10,11,12].

Several conventional surfacing techniques, such as high-energy laser beam [13,14,15], plasma spraying [16,17], cast sinter [18], and electron beam irradiation [19,20], have been developed to fabricate surface metal matrix composites. However, it should be pointed out that these techniques are generally based on liquid phase processing at high temperatures. In this case, it is hard to avoid the excessive interfacial reactions of added magnetic powder with aluminum matrix and the resulting formation of some undesirable phases, which weaken the bonding strength between them, as well as the magnetization property [21]. Obviously, if the processing of surface composite is carried out in the solid state, most of the interfacial reactions between the fillers and the matrix can be prevented, or at least depressed [21,22].

Friction stir processing (FSP) has been suggested as a potential technique to produce a magnetic surface layer on a pure aluminum surface for a number of reasons. The most important one arises from the fact that it is a solid state process. Therefore, the problem related to liquid phase processing can be eliminated [23]. Moreover, the rapid heating and cooling of the friction stir processing (FSP) reduces the possibility of the formation of undesirable interfacial reactions.

In the present work, we report the fabrication of a new aluminum alloy with magnetic properties by dispersing iron and magnetite particles in a surface layer of aluminum (Al) 1050-H24 alloy using FSP.

## 2. Experimental Procedures

Commercially pure aluminum (Al-1050-H24) plates, 5 mm in thickness, were used as the base material. A friction stir processing machine (vertical type with rigid column, Hitachi, Tokyo, Japan), equipped with a tool of steel SKD61 with a shoulder of a 14 mm diameter and a square probe of 5 mm in diagonal length and 3.3 mm height, was used to perform the FSP. The dispersed magnetic particles were pure Fe and pure Fe_3_O_4_ with average particles size of 4 µm and 180 µm, respectively. The dispersed particles were packed in a groove, 3 mm in width and 1.5 mm in depth, cut on the Al plate. The FSP tool was rotated at a rate ranging from 1000 to 1500 rpm for Fe particles and from 1000 to 2000 rpm for Fe_3_O_4_ particles, and travelled at a speed of 1.66 mm/s. Double and triple passes were applied in order to improve the homogeneity of the dispersed particle distribution. In the second pass, the tool was travelled along the same line as the first and third ones, but in the opposite direction. This means that the advancing side of the first and third passes became the retreating side in the second pass.

The microstructure study was carried out using an optical microscope (Olympus optical microscope with digital camera, Tokyo, Japan) and a scanning electron microscope (FE-SEM, ELIONIX, ERA-8800FE) equipped with an energy dispersive X-ray spectroscopy (EDS), JEOL, Tokyo, Japan). Additionally, The samples were analyzed with an X-ray diffractometer (XRD) (D8 Discover with a GADDS system, 35 kv, 80 mA, MoKα radiation, Bruker Corporation, Karlsruhe, Germany) to identify the phases that were originally found or formed inside the FSP nugget zone after triple passes. The microhardness was measured with AKASHI Model Vickers hardness tester (Akashi Corporation, Akashi-shi, Hyogo, Japan) at a 200 g load applied for 15 s through the mid-plane of the cross-section of the nugget of each condition. The hardness distribution was measured along three parallel lines that were 1–2 mm deep from the upper surface, and separated by a distance of 0.5 mm. The interval between the indentations on the same line was 0.75 mm. All measured data along the three lines were collected and averaged for each condition. The magnetic properties of the resulted nugget zone were investigated using superconducting quantum interference device (SQUID) magnetometer (MPMS XL, Quantum Design, San Diego, CA, USA), at a temperature of 5 K, and at a magnetic field ranging from +20 kOe to −20 kOe. The test was carried out on samples with the dimensions of about 10 mm × 3 mm × 1 mm, cut approximately from the center of the nugget zone fabricated by the triple FSP passes. The magnetization hysteresis loops as a function of the applied magnetic field were recorded automatically, based on the device software.

## 3. Results and Discussion

### 3.1. Macro and Microstructure of the Nugget Zone

#### 3.1.1. Fe Powder

**Figure 1:**
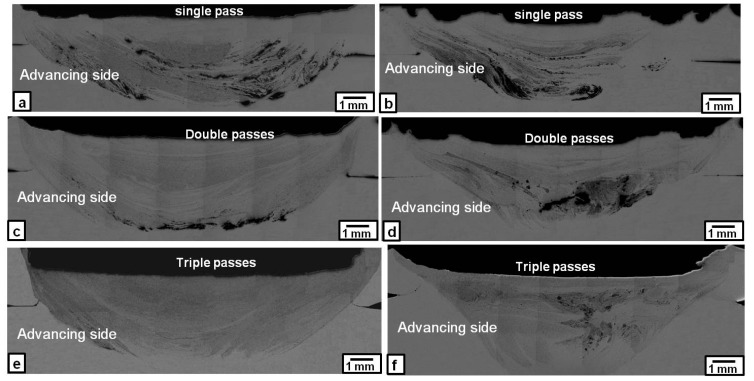
Macrographs of the Fe-particles dispersed nugget zone produced by single, double and triple friction stir processing (FSP) passes at following rotation speeds: (**a**,**c**,**e**) 1000 rpm; and (**b**,**d**,**f**) 1500 rpm.

The Microstructure of the nugget zones produced by first, second, and third FSP passes at rotation speeds of 1000 and 1500 rpm are shown in Figure 1. In general, the distribution of the Fe particles (grey contrast) was improved by increasing the number of passes. At a rotation speed of 1000 rpm, some of the Fe particles, after the first FSP pass, as shown in Figure 1a, were agglomerated into clusters or banded clusters (black contrast) in the nugget zone. By applying the second FSP pass, the Fe particles were distributed more widely and uniformly in the nugget zone, except for some Fe clustering located at the bottom of the nugget zone, as shown in Figure 1c. To improve the homogeneity of the Fe particles and eliminate the clustering in the nugget area, a third FSP pass was applied, resulting in an almost homogenous distribution of Fe particles in the nugget zone area without any observable cracks or large defects, as shown in Figure 1e. This is due to the stirring action generated in every pass with the rotated tool. On the other hand, when the rotation speed was increased to 1500 rpm, the homogeneity of the Fe particles within the nugget zone became poorer than that obtained at the 1000 rpm rotation speed. As shown in Figure 1b,d,f, high density iron areas (dark grey) were detected in the center of the nugget zone, even after the third pass at 1500 rpm.

**Figure 2:**
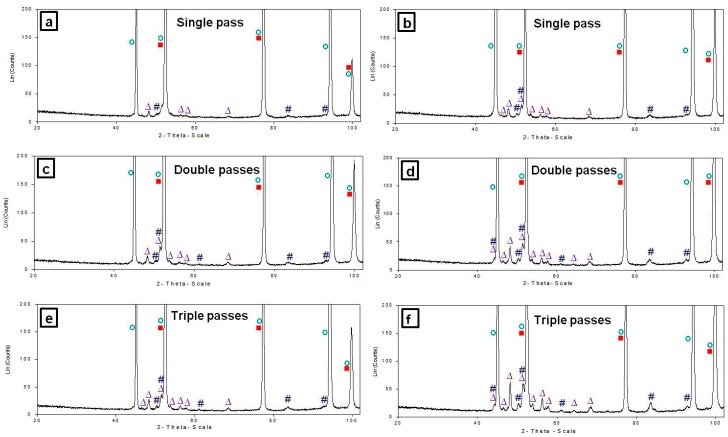
XRD patterns of nugget zones cross-sections (Al-Fe) after single, double and triple FSP passes at following rotation speeds: (**a**,**c**,**e**) 1000 rpm; and (**b**,**d**,**f**) 1500 rpm. (○ Al, ■ Fe, ∆ Al_3_Fe, # Al_5_Fe_2_).

X-ray diffraction (XRD) patterns obtained from the cross-sections of the nugget zones produced by the first, second and third passes at rotation speeds of 1000 and 1500 rpm are shown in Figure 2. All of the observed nugget zones consisted of mixtures of Al and Fe. Furthermore, small reflection peaks of the Al_3_Fe phase were detected, even after the first FSP pass, along with very small reflection peaks of the Al_5_Fe_2_ phase.

**Figure 3:**
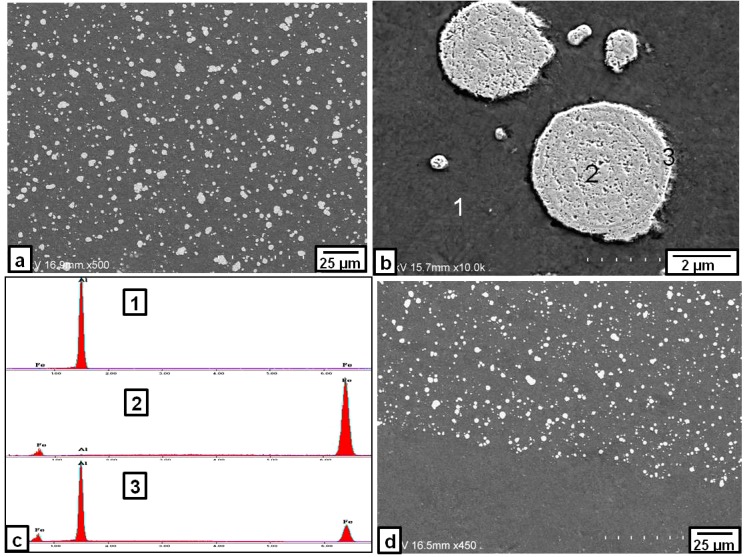
SEM image of the Fe particles dispersed in the nugget zone produced with triple FSP passes at a rotation speed of 1000 rpm: (**a**) nugget zone center; and (**b**) higher magnification of some Fe dispersed particles; (**d**) nugget zone bottom; and (**c**) the EDS spectra for point 1, 2 and 3 in (**b**).

SEM microstructures observed in the nugget zone after the third pass at a rotation speed of 1000 rpm are shown in Figure 3. The Fe particles (white and round in shape) were distributed almost homogenously in the Al matrix without any cracks or voids, as shown in Figure 3a. The ratio of the Fe particles in the nugget zone relative to the Al matrix was about 17%. A magnified image of relatively larger dispersed Fe particles in the Al matrix, as shown in Figure 3b, suggested the formation of intermetallic compounds only in the narrow area, close to the edge of the round particle, while the inner portion of the particle remained as un-reacted Fe, as confirmed by the EDS analyses in Figure 3c. The boundary between the nugget zone and the remaining Al plate appeared to be well bonded without any defects, as shown in Figure 3d.

**Figure 4:**
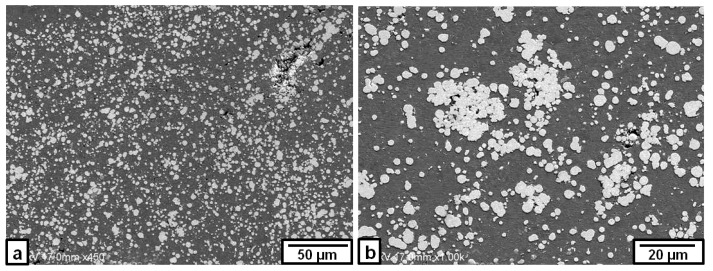

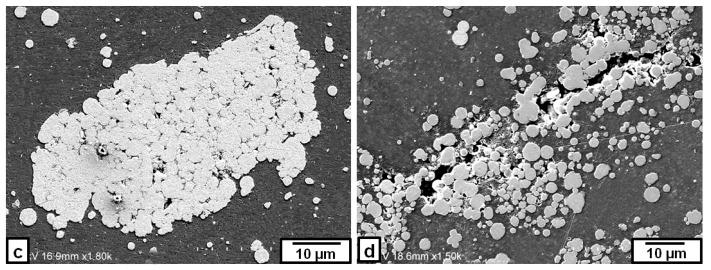
SEM image of the Fe particles dispersed in some areas produced by triple FSP passes at a rotation speed of 1500 rpm: (**a**) nugget center; (**b**) area involving Fe particles of high density; (**c**) Fe particles cluster; and (**d**) cracks in an area involving Fe particles of high density.

When the rotation speed was increased to 1500 rpm, there were some inhomogeneities of the Fe particle distributions in the micro-scale within the nugget zone, even after the third pass, as shown in Figure 4a. Some iron particles tended to be agglomerated to form clusters, as shown in Figure 4b,c. Moreover, some micro-cracks were observed in areas where the Fe particles are densely concentrated, as shown in Figure 4d.

**Figure 5:**
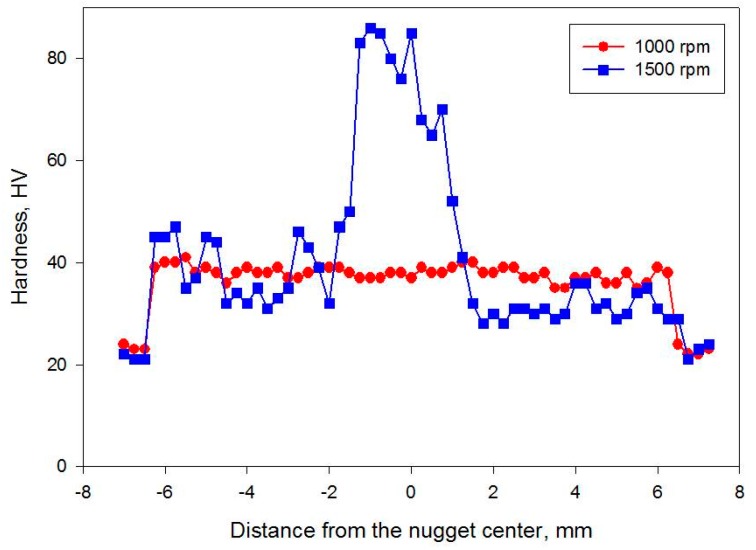
Hardness distributions in the horizontal section of the Fe nugget zone fabricated by triple passes at rotation speeds of 1000 and 1500 rpm.

The hardness profiles of the nugget zone cross-section produced at rotation speeds of 1000 and 1500 rpm by the triple FSP passes are presented in Figure 5. At a rotation speed of 1000 rpm, the hardness was relatively uniformly distributed around 38 HV in the nugget zone, suggesting a relatively homogenous microstructure within the nugget zone. In contrast, it showed a large scattering within the nugget zone when the rotation speed was increased to 1500 rpm, in accordance with the iron particle distribution observed in the macrographs shown in Figure 1.

#### 3.1.2. Fe_3_O_4_ Powder

**Figure 6:**
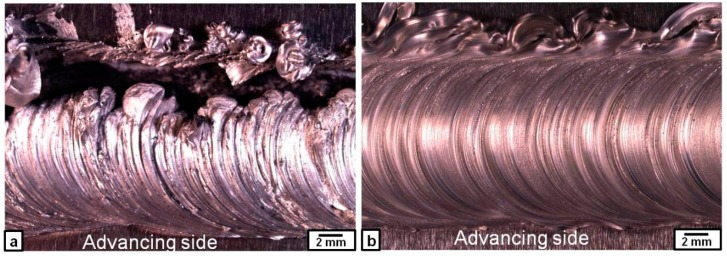
Macrographs of the top view of the nugget zone involving Fe_3_O_4_ particles fabricated at rotation speeds of (**a**) 1000 rpm and (**b**) 1500 rpm.

**Figure 7:**
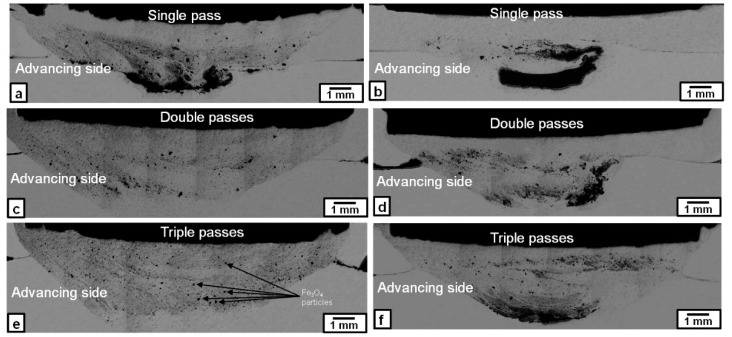
Macrographs of the Fe_3_O_4_ particles distribution in the nugget zone produced by single, double and triple FSP passes at rotation speeds of 1500 rpm (**a**,**c**,**e**) and 2000 rpm (**b**,**d**,**f**).

When the Fe_3_O_4_ particles were dispersed, the rotation speed of 1000 rpm was not enough to obtain a sound nugget; i.e., the nugget zone was broken on the retreating side to large debris of mixture of aluminum and Fe_3_O_4_ particles, leaving a longitudinal empty gap along the travelling line as shown in Figure 6a, while, at 1500 rpm, the nugget zone was almost sound (Figure 6b). At rotation speeds not less than 1500 rpm, the nugget zones with dispersed Fe_3_O_4_ particles exhibited similar features to those observed when the Fe particles were dispersed, as shown in Figure 7. At a rotation speed of 1500 rpm, the Fe_3_O_4_ particles were distributed in most of the nugget zone areas towards the advancing side after the first pass, except some clustering of Fe_3_O_4_ particles in the bottom of the nugget zone, as shown in Figure 7a. By applying triple FSP passes, the Fe_3_O_4_ particles were relatively uniformly dispersed in most of the nugget zone area without any observable cracks or macro-defects, as shown in Figure 7e. The relatively large black spots scattered in the nugget zone after triple FSP passes were Fe_3_O_4_ particles of their original size (~180 µm). However, at a rotation speed of 2000 rpm, inhomogeneous distribution of the Fe_3_O_4_ particles and its agglomeration were apparently observed after the first and second, and even third, passes. After the first pass, as shown in Figure 7b, the Fe_3_O_4_ particles were distributed in a clear “C” shape area in the center of the nugget zone, leaving the rest of the nugget zone at a much lower dispersed particles density. Applying triple passes at the same rotation speed of 2000 rpm changed the distribution shape of the Fe_3_O_4_ particles; they were concentrated at the bottom of the nugget zone with a high density and in the upper part, parallel to the shoulder surface, but at a lesser density, leaving the rest of the nugget zone at much lower densities of Fe_3_O_4_ particles.

**Figure 8:**
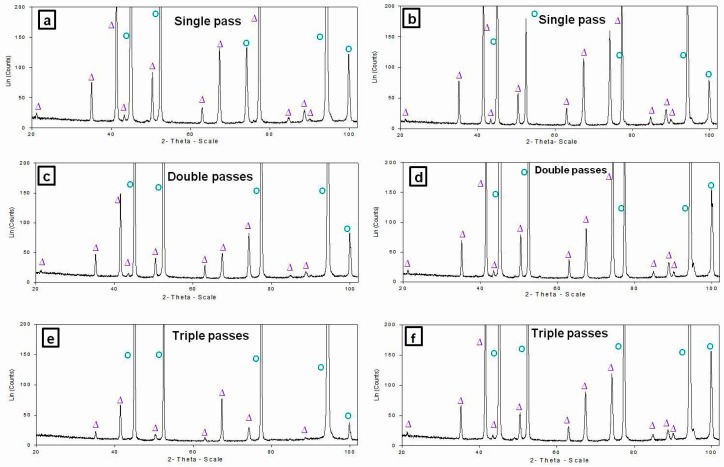
XRD patterns of nugget zones cross-sections (Al-Fe_3_O_4_) after single, double and triple FSP passes at rotation speeds of 1500 rpm (**a**,**c**,**e**) and 2000 rpm (**b**,**d**,**f**) (○ Al and ∆ Fe_3_O_4_).

The XRD patterns from the nugget zone cross-sections after the first, second and third FSP passes, at rotation speeds of 1500 and 2000 rpm, are shown in Figure 8. It was clear that there were no new phases except the added Fe_3_O_4_ and the Al matrix. This suggests that almost no reaction occurred between the Fe_3_O_4_ particles and the Al matrix during the three FSP passes.

**Figure 9:**
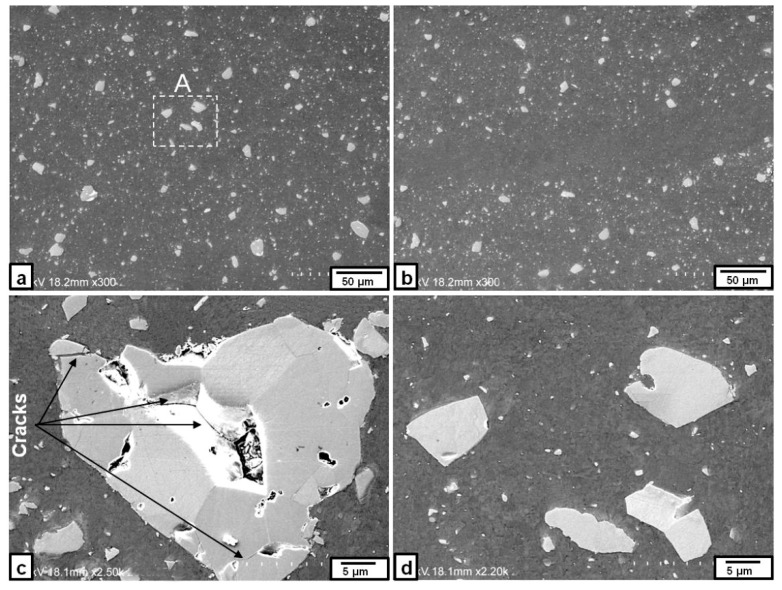
SEM images of the Fe_3_O_4_ particles dispersed in the nugget zone produced by triple FSP passes at a rotation speed of 1500 rpm: (**a**) central area; (**b**) banded structure in the center; (**c**) large Fe_3_O_4_ particle involving cracks; and (**d**) magnified image of area A in (**a**).

Microscopically, the SEM images of the nugget cross-section produced by triple FSP passes at a rotation speed of 1500 rpm, as shown in Figure 9a, revealed that the Fe_3_O_4_ particle distribution in the matrix was almost uniform in most of the nugget zone without any observable defects. In some areas in the center of the nugget zone, the Fe_3_O_4_ particles showed some banded structures, as shown in Figure 9b. These SEM images show the presence of very fine Fe_3_O_4_ particles (few micrometers in size), which is much smaller than the as-received Fe_3_O_4_ particles (~180 µm). This may be explained as a result of the stirring effect of the tool which can break the particles into small or fine parts. This explanation was confirmed by the cracks, which were detected inside and near the edge of large Fe_3_O_4_ particle, as shown in Figure 9c. A higher magnification of the selected area (A) in Figure 9a suggests that the Fe_3_O_4_ particles had clean surfaces and were tightly bonded to the aluminum matrix (see Figure 9d).

**Figure 10:**
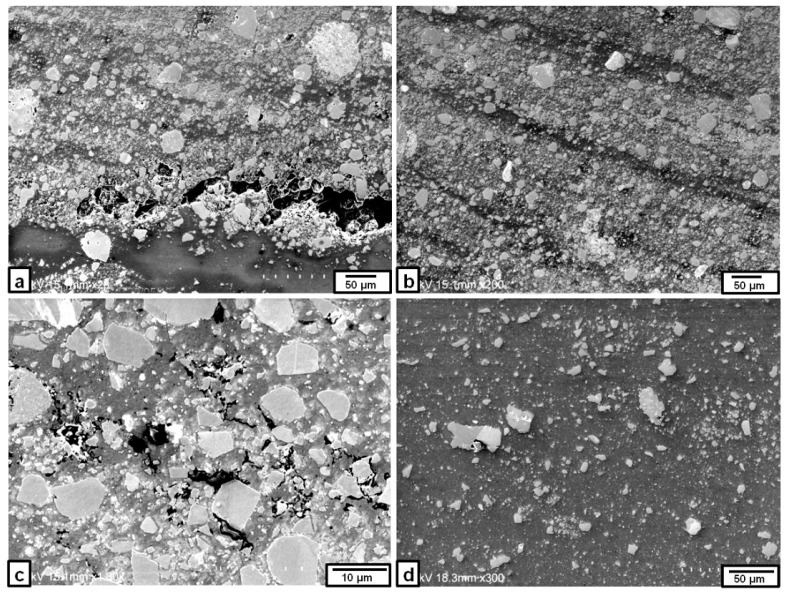
SEM images of the Fe_3_O_4_ particles dispersed in some areas of the nugget zone produced by triple FSP passes at a rotation speed of 2000 rpm: (**a**,**b**,**c**) nugget bottom; and (**d**) nugget upper portion.

Figure 10 shows some local areas in the nugget zone fabricated by triple FSP passes at a rotation speed of 2000 rpm. The Fe_3_O_4_ particles were clustered at the bottom of the nugget zone, accompanied by void-like cracks, as shown in Figure 10a. In addition, some Fe_3_O_4_ particles tended to agglomerate in a banded structure towards the advancing side, as shown in Figure 10b. A close up of these areas, as shown in Figure 10c, indicated that there were some cracks propagated between Fe_3_O_4_ particles. Regarding the upper part of the nugget zone, the Fe_3_O_4_ particles also exhibited inhomogeneous distribution parallel to the upper surface, as shown in Figure 10d.

**Figure 11:**
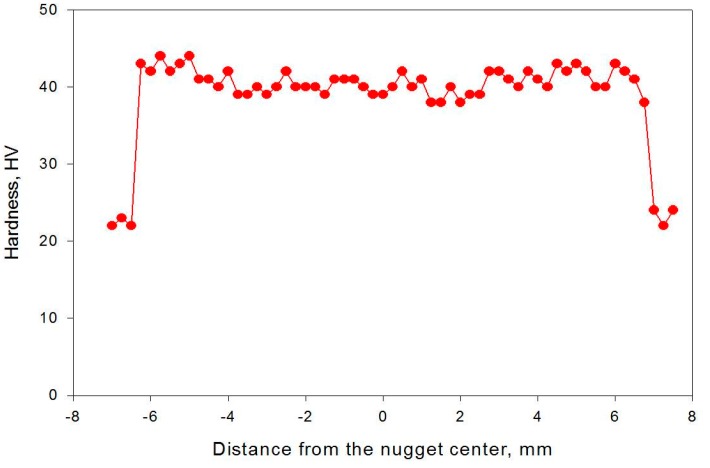
Hardness distribution in the horizontal section of the Fe_3_O_4_-nugget zone fabricated by triple FSP passes at a rotation speed of 1500 rpm.

Similar to Fe, the hardness profile of the nugget zone containing Fe_3_O_4_ particles fabricated by triple FSP passes at a rotation speed of 1500 showed a reasonable distribution around 41 HV, which confirmed the microstructure analysis, as observed in Figure 11.

### 3.2. Magnetic Properties of the Nugget Zone

**Figure 12:**
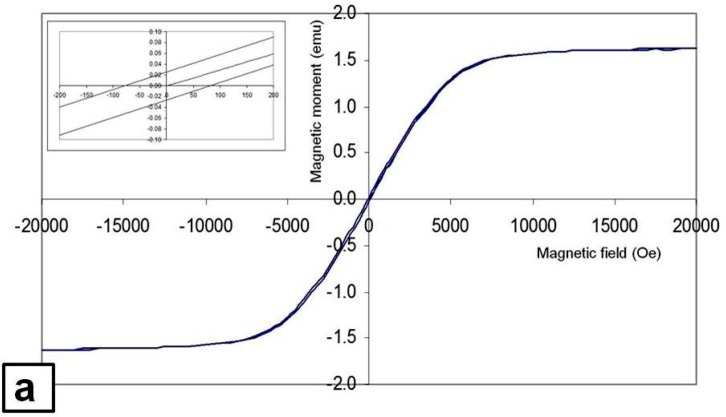

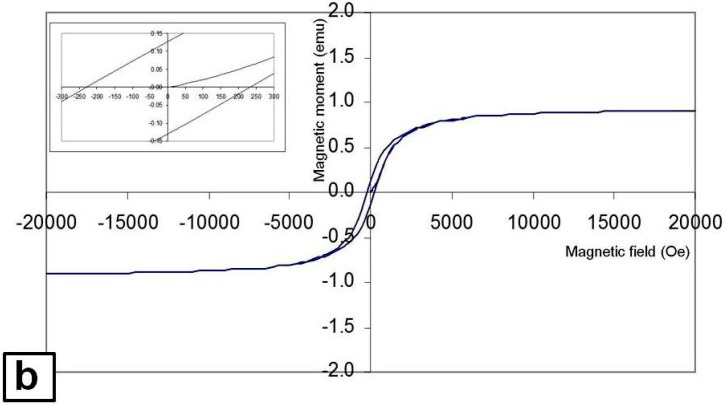
Magnetization hysteresis loops for nugget zone contained dispersed: (**a**) Fe; and (**b**) Fe_3_O_4_ particles through FSP.

The magnetization hysteresis loops obtained from the nugget zones containing Fe and Fe_3_O_4_ particles dispersed by triple FSP passes at rotation speeds of 1000 rpm (for Fe) and 1500 (for Fe_3_O_4_) are shown in Figure 12. The loops for both nugget zones exhibited a typical magnetic material feature; the magnetic moment rose with the applied magnetic field until it reached a saturation magnetization at a magnetic field of about 7 kOe for the nugget zone with the Fe particles, and 5 kOe for the nugget zone with the Fe_3_O_4_ particles, which are much lower than that required for aluminum alloys without magnetic particles (100 kOe [24]), and very close to those of the ferromagnetic and ferrimagnetic materials. Moreover, the magnetic susceptibilities, 0.2 for the Fe-dispersed nugget zone and 0.016 for the Fe_3_O_4_-dispersed nugget zone, were much higher than those of the aluminum alloys (2.07 × 10^−5^ [24]). These results suggest that the nugget zones into which the magnetic particles were introduced using FSP had magnetic properties.

Table 1 summarizes the measured saturation magnetic moment, saturation magnetization (Ms) and the coercivity (Hc) for the nugget zone involving the dispersed particles of Fe and Fe_3_O_4_. It was clear that the saturation magnetization of the nugget zone containing the Fe particles was higher than that of the nugget zone with Fe_3_O_4_ particles, whereas the coercivity of the former was much lower than that of the later.

Since aluminum is non-magnetic material, the large saturation magnetization of the Fe-dispersed nugget zone comes from the iron particles. The saturation magnetization, 33 emu/g, is almost 15% of that of pure iron (magnetization of pure iron ~221.5 emu/g at T = 5 K). This ratio was slightly smaller than the content of the dispersed Fe particles (17%), which may be due to the iron portion reacted with aluminum matrix during FSP. On the other hand, the saturation magnetization of the Fe_3_O_4_-dispersed nugget zone was 15 emu/g, which is almost 16.3% of that of magnetite (92 emu/g).

The low coercivity of the Fe-dispersed nugget zone is useful in the soft magnetic applications, where high permeability is required. In this field, the FSP, having a refining effect, may be considered as a promising tool to fabricate a soft super magnetic material, since decreasing the particle size of magnetic material to below a critical size (an order of 10 nm), decreases the coercivity to zero. These very fine ferromagnetic particles have short relaxation times and behave supermagnetically.

## 4. Conclusions

The friction stir processing is used to fabricate a surface composite layer on the aluminum plate by dispersing iron and magnetite particles. The iron and magnetite magnetic powders were packed in a groove of 3 mm in width and 1.5 mm in depth, cut on the aluminum surface. The tool rotated at a rate of 1000–2000 rpm, and moved along the groove at a travelling speed of 1.66 mm/s. Double and triple passes were applied. The results obtained can be summarized as follows:

- The iron particles distributed almost homogenously in the nugget zone at only a rotation speed of 1000 rpm after triple FSP passes. Limited interfacial reactions occurred between the iron particles and aluminum matrix to form thin intermetallics (Al_3_Fe and Al_5_Fe_2_) between them.
- The rotation speed of 1000 rpm was not enough to form a sound nugget when the reinforced particles were Fe_3_O_4_, while the Fe_3_O_4_ particles were dispersed homogenously in a sound nugget zone when the rotation speed was increased to 1500 rpm. No reaction products could be detected between the Fe_3_O_4_ particles and the aluminum matrix.
- The saturation magnetization (Ms) of the Fe-dispersed nugget zone was higher than that of the Fe_3_O_4_-dispersed nugget zone. Moreover, there were good agreement between the obtained saturation magnetization values relative to that of pure Fe and Fe_3_O_4_ materials and the volume content of the dispersed particles in the nugget zone

## Figures and Tables

**Table 1:** The measured magnetic properties for the Fe and Fe_3_O_4_ metal matrix composites nugget zone after triple friction stir processing (FSP) passes.

Material	Magnetic Moment (emu)	Saturation Magnetization (emu/g)	Coercivity (Oe)
Fe-dispersed MMCs	1.6	33	82.7
Fe_3_O_4_-dispersed MMCs	0.93	15	228.9
